# Anhedonia in schizophrenia and major depression: state or trait?

**DOI:** 10.1186/1744-859X-8-22

**Published:** 2009-10-08

**Authors:** Lorenzo Pelizza, Alberto Ferrari

**Affiliations:** 1 Guastalla Psychiatric Service, Reggio Emilia Mental Health Department, Reggio Emilia, Italy

## Abstract

**Background:**

In schizophrenia and major depressive disorder, anhedonia (a loss of capacity to feel pleasure) had differently been considered as a premorbid personological trait or as a main symptom of their clinical picture. The aims of this study were to examine the pathological features of anhedonia in schizophrenic and depressed patients, and to investigate its clinical relations with general psychopathology (negative, positive, and depressive dimensions).

**Methods:**

A total of 145 patients (80 schizophrenics and 65 depressed subjects) were assessed using the Physical Anhedonia Scale and the Social Anhedonia Scale (PAS and SAS, respectively), the Scales for the Assessment of Positive and Negative Symptoms (SAPS and SANS, respectively), the Calgary Depression Scale for Schizophrenics (CDSS), and the Hamilton Depression Rating Scale (HDRS). The statistical analysis was performed in two steps. First, the schizophrenic and depressed samples were dichotomised into 'anhedonic' and 'normal hedonic' subgroups (according to the 'double (PAS/SAS) cut-off') and were compared on the general psychopathology scores using the Mann-Whitney Z test. Subsequently, for the total schizophrenic and depressed samples, Spearman correlations were calculated to examine the relation between anhedonia ratings and the other psychopathological parameters.

**Results:**

In the schizophrenic sample, anhedonia reached high significant levels only in 45% of patients (n = 36). This 'anhedonic' subgroup was distinguished by high scores in the disorganisation and negative dimensions. Positive correlations of anhedonia with disorganised and negative symptoms were also been detected. In the depressed sample, anhedonia reached high significant levels in only 36.9% of subjects (n = 24). This 'anhedonic' subgroup as distinguished by high scores in the depression severity and negative dimensions. Positive correlations of anhedonia with depressive and negative symptoms were also been detected.

**Conclusion:**

In the schizophrenic sample, anhedonia seems to be a specific subjective psychopathological experience of the negative and disorganised forms of schizophrenia. In the depressed sample, anhedonia seems to be a specific subjective psychopathological experience of those major depressive disorder forms with a marked clinical depression severity.

## Background

"Pleasure is the alpha and omega of a happy life"

(Epicurus: 'Letter to Menoeceus') [1].

Anhedonia, a term first used by Ribot [2] in 1896, is a diminished capacity to experience pleasure. It describes the lack of interest and the withdrawal from all usual pleasant activities [3,4]. Chapman *et al*. [5] defined two different types of hedonic deficit: physical anhedonia and social anhedonia. Physical anhedonia represents an inability to feel physical pleasures (such as eating, touching and sex). Social anhedonia describes an incapacity to experience interpersonal pleasure (such as being and talking to others).

### Anhedonia and schizophrenia

Since the writings of Bleuler [6] and Kraepelin [7], anhedonia has figured in clinical descriptions of the 'core' deficits of schizophrenia. Today, it is still commonly included by many authors [8-15] in the negative symptomatology of schizophrenic disorders. For example, Andreasen [10] has inserted the hedonic deficit into the diagnostic criteria for the 'negative syndrome' of schizophrenia, defining a specific 'anhedonia/asociality' subscale in the Scale for the Assessment of Negative Symptoms (SANS). Carpenter *et al*. [11] also considered anhedonia as a 'primary' and 'enduring' negative feature of the 'deficit syndrome' of schizophrenia. In their Schedule for Deficit Syndrome (SDS) [13], the hedonic inability concerned at least three of the six items proposed ('restricted emotional range', 'curbing of interests' and 'diminished social drive'). In a 10-year follow-up study, Herbener and Harrow [15] have shown that anhedonia was a stable clinical feature of the schizophrenic course and a distinctive state-like symptom of schizophrenic chronicity.

Contrary to the hypothesis of anhedonia as a 'core' symptom of schizophrenic disorders, other authors [16-19] considered the hedonic deficit as a marker of genetic vulnerability to schizophrenia, and either a contributing or potentiating personological factor for the development of schizophrenic illness. For example, Rado [17] has suggested that anhedonia was a main genetically transmitted defect both in overt schizophrenia and in compensated schizotypal subjects. Some years later, Meehl [18] integrated Rado's view into a theory of neurological dysfunction in schizophrenic disorder, positing that anhedonia was a 'cardinal' enduring trait preceding and possibly causing schizophrenia. More recently, several authors [20-24] have found that individuals with deviantly high scores on the Chapman Anhedonia Scales were disproportionately more likely to develop psychotic-like experiences and schizophrenia spectrum disorders. Schurhoff *et al*. [24] considered those psychotic subjects as a distinct familial subtype of schizophrenia, characterised by a highly anhedonic first-degree relatives and a threefold familial risk of schizophrenia spectrum disorders.

### Anhedonia and depression

Since the writings of Clouston [25], Bevan-Lewis [26] and Kraepelin [7], anhedonia had figured as a main symptom in clinical descriptions of 'melancholia'. Today, it is still commonly included by many authors [27-32] among the 'nuclear' symptoms of major depressive disorder. For example, Van Praag [27] has inserted the hedonic deficit into his 'vital syndrome' definition and Klein [28] has used the term 'endogenomorphic' to describe a distinct subtype of major depression with a marked anhedonic symptomatology. Fawcett *et al*. [29] also suggested that in this endogenomorphic depressed subgroup (characterised by the lack of responsiveness to pleasure) the anhedonic feature had to be considered as a post-depressive 'scar' symptom.

According to Klein's position, the American Psychiatric Association (APA) [30] has assigned a central role to anhedonia in the Diagnostic and Statistical Manual, fourth edition text revision (DSM-IV-TR) definition of 'major depressive episode' and in its 'melancholic features' specification. In the same way, in the International Classification of Diseases, 10th revision (ICD-10), the World Health Organization (WHO) [31] has resolved to include curbing of interests and the incapacity to feel pleasure and to experience pleasant emotions among the 'biological symptoms' of major depression. More recently, Joiner *et al*. [32] also found that patients with major depressive disorder presented higher scores on Beck Depression Inventory (BDI) anhedonic items [33] than schizophrenic subjects, suggesting that anhedonia was a specific state-like feature of depressive illness, which was clinically related to marked psychomotor retardation [34] and recurrent suicidal ideation [35].

Contrary to the hypothesis of anhedonia as a 'nuclear' symptom of major depression, other authors [36-39] have considered the hedonic deficit as a marker of genetic vulnerability to major depressive disorder, and either a contributing or potentiating personological factor for the development of depressive illness. For example, Meehl [37] has used the term 'hedonic capacity' to describe a positive psychological attribute of personality which presented a 'normal' distribution in general population. In his opinion, anhedonia has to be considered a constitutional (genetically transmitted) enduring trait that preceded and possibly caused an endogenous depression. Some years later, Akiskal and Weise [38] included the hedonic deficit among the basic features of 'depressive temperament' (together with sadness, pessimism, introversion, passivity, and anxiety). Moreover, Loas [39] proposed a 'vulnerability to depression model' centred on anhedonia. In his opinion, an interaction (during adolescence and/or adulthood) between a constitutional hedonic inability and negative psychosocial stressful events caused the development of an endogenomorphic (unipolar) depression.

In the last two decades, anhedonia has also been described in Parkinson disease [40] and in other different axis I disorders, particularly drug abuse [41-43]. According to Martinotti *et al*. [42], the frequent presence of hedonic deficit in alcohol and substance use disorders is significant in relation to the high prevalence of those disorders in schizophrenia and major depression.

In summary, there have been contradictory data regarding the relationship between anhedonia and the clinical symptoms of schizophrenia and major depression [44]. Therefore, the aims of this study were to examine psychopathological features of anhedonia in schizophrenics and depressed patients, and investigate its clinical relationship with diagnostic dimensions (positive, negative, disorganised, and depressive symptoms) of schizophrenia and major depressive disorder. Moreover, this study aimed to elucidate the nature of anhedonia as either state-like or trait-like feature in general schizophrenic and depressive psychopathology.

## Methods

### Sampling

A series of consecutive DSM-IV-TR schizophrenic and depressed outpatients, attending the Guastalla Psychiatric Service (Reggio Emilia Mental Health Department) for maintenance treatment were assessed. A total of 145 subjects (80 schizophrenics and 65 depressed patients) were selected from within a larger cohort of chronic psychotic and depressed patients, from which substance abusers, illiterate patients, markedly cognitively deteriorated patients, grossly non-compliant patients, and those suffering from mental retardation or organic mental disorders were excluded.

According to DSM-IV-TR criteria [30], 30 (37.5%) schizophrenic subjects were diagnosed as paranoid, 28 (35%) as residual, 14 (17.5%) as disorganised, and 8 (10%) as catatonic schizophrenia subtype. Their sociodemographic data are shown in Table 1. Of the analysed psychotic patients, 46 (57.5%) were men and 34 women (42.5%). Only 24 (30%) were married and 36 (45%) were working during the evaluation time. Their ages ranged between 18 and 50 years (mean ± standard deviation (SD) = 36.21 ± 9.36). They attended school for a range of 4 to 16 years (10.85 ± 3.34) and the average number of years since the onset of illness was 11.57 ± 7.95.

**Table 1:** Sociodemographic data and anhedonia scores of the total sample (n = 145 patients)

**Sociodemographic variables**	**Schizophrenic patients (n = 80)**	**Depressed patients (n = 65)**
Gender:		
Male	46 (57.5%)	30 (46.2%)
Female	34 (42.5%)	35 (53.8%)
Civil state:		
Unmarried	56 (70%)	31 (47.7%)
Married	24 (30%)	34 (52.3%)
Occupation:		
Employed	36 (45%)	37 (56.9%)
Unemployed	44 (55%)	28 (43.1%)
Age (years)	36.21 ± 9.36	35.54 ± 8.24
Duration of illness (years)	11.57 ± 7.95	10.63 ± 6.44
Education (years)	10.85 ± 3.34	11.08 ± 2.67
PAS total score	20.90 ± 8.04	15.32 ± 6.72
SAS total score	15.87 ± 6.35	13.07 ± 5.66
PAS cut-off (≥ 18)	48 (60%)	27 (41.5%)
SAS cut-off (≥ 12)	52 (65%)	32 (49.2%)
'Double cut-off'	36 (45%)	24 (36.9%)

Mean ± standard deviation (SD) or percentages (%) are reported.

According to DSM-IV-TR criteria [30], 28 (43.1%) depressed subjects were diagnosed as 'major depressive disorder: single episode' and 37 (56.9%) as 'major depressive disorder: recurrent' subtype. Their sociodemographic data are shown in Table 1. Of the analysed depressed patients, 35 (53.8%) were women and 30 men (46.2%). Only 34 (52.3%) were married and 37 (56.9%) were working during the evaluation time. Their ages ranged between 19 and 47 years (35.54 ± 8.24). They attended school for a range of 5 to 18 years (11.08 ± 2.67) and the average number of years since the onset of illness was 10.63 ± 6.44. All the psychotic and depressed patients gave their written informed consent to the psychopathological assessment.

### Psychopathological assessment

General psychopathology was assessed using the Scales for the Assessment of Positive and Negative Symptoms (SAPS and SANS) [45], the Calgary Depression Scale for Schizophrenics (CDSS) [46], and the Hamilton Depression Rating Scale (HDRS) [47], in order to obtain a global picture of depressive symptoms and positive, disorganised, and negative psychotic dimensions, according to the factorial tripartite models of Liddle [48] and Andreasen and Arndt [49].

Anhedonia was assessed using the scales proposed by Chapman *et al*. (Scales for Physical and Social Anhedonia (PAS and SAS, respectively)) [5], which are two 'true/false' self-report instruments measuring the personological (enduring trait-feature) diminished ability to experience sensory and interpersonal pleasures (such as eating, touching, being and talking to others, sex, smell, and sound). Regarding the PAS and SAS cut-offs above which a subject can be categorised as 'anhedonic', we decided to use the values proposed by the French versions of the Chapman scales (respectively, ≥ 12 for social anhedonia and ≥ 18 for physical anhedonia) [50], because of their higher specificity and sensitivity than Chapman's original limits [51]. To select a 'really anhedonic' (schizophrenic or depressed) subgroup, we also preferred to use the 'double (PAS and SAS) cut-off', according to which the subjects had to reach both PAS and SAS cut-off at the same time. Differently, the SANS 'anhedonia/asociality' subscale must be considered as a symptomatological complex (state-like feature) indicating the individual hedonic state deficit in pleasant activities [10].

To obtain a thorough evaluation, data were collected on the same day for each patient. All subjects were interviewed at the time of their admission by two clinicians of the Guastalla Psychiatric Service. Calibration meetings to ensure that ratings remained stable over time and rater drift did not occur were performed throughout the data collection phase for each of the interview-based scales (SAPS, SANS, CDSS, HDRS).

### Data analysis

The statistical analysis of the data was performed in two steps. At first, both the schizophrenic and depressed samples were dichotomised into 'anhedonic' and 'normal hedonic' subgroups, using the 'double cut-off'. Then, they were compared on the general psychopathology scales (negative, positive, disorganised, and depression dimension scores) using the Mann-Whitney Z test. Subsequently, both for the total schizophrenic and depressed sample, Spearman correlations were calculated to examine the possible relation between general psychopathological parameters and Chapman anhedonia ratings (PAS and SAS total scores).

## Results

### Schizophrenic patients

The mean anhedonia scores for the schizophrenic sample were 20.90 ± 8.04 for physical anhedonia (PAS total score) and 15.87 ± 6.35 for social anhedonia (SAS total score) (Table 1). For the analysed schizophrenics, 48 (60%) reached or passed the PAS cut-off, 52 (65%) the SAS cut-off and 36 (45%) the 'double cut-off' (Table 1).

The comparison for general psychopathological parameters between 'anhedonic' and 'normal hedonic' schizophrenic subgroups revealed that the former displayed higher levels of negative symptoms (SANS total score (*P *< 0.05)) and disorganisation (*P *< 0.05) (particularly in the SAPS 'formal thought disorders' subscale score (*P *< 0.01)). No differences in positive dimension and depressive symptoms were observed (Table 2). No differences were detected between schizophrenic subgroups in terms of gender, civil state, occupation, age, years of education, duration of illness, type and dosage of medication (typical vs atypical antipsychotic drugs).

**Table 2:** Comparison of general psychopathological parameters between 'anhedonic' and 'normal hedonic' schizophrenics

**Psychopathological variables**	**'Normal hedonic' schizophrenics (n = 44)**	**'Anhedonic' schizophrenics (n = 36)**	**Z value**
Negative dimension (SANS total score)	32.35 ± 11.63	37.86 ± 11.41	-2.69*
Affective flattening	9.45 ± 6.58	12.00 ± 6.57	-1.84
Alogia	3.25 ± 3.03	3.60 ± 2.47	-0.31
Avolition/apathy	7.94 ± 2.95	9.00 ± 2.85	-1.67
Anhedonia/asociality	12.00 ± 3.73	13.26 ± 3.38	-0.79
Positive dimension	10.61 ± 10.43	10.82 ± 9.64	-0.14
Hallucinations	3.64 ± 5.96	3.41 ± 4.99	0.19
Delusions	6.96 ± 6.53	7.41 ± 7.29	-0.78
Disorganised dimension	8.61 ± 6.41	12.89 ± 10.58	-2.67*
Bizarre behaviour	1.32 ± 2.32	2.20 ± 3.01	-1.41
Formal thought disorders	1.03 ± 1.97	4.82 ± 7.22	-3.14**
Attentional impairment	2.03 ± 2.34	2.56 ± 2.80	-0.55
Depression (CDSS total score)	3.87 ± 4.11	4.23 ± 3.57	-0.69

Mean ± standard deviation (SD) and Mann-Whitney Z test values are reported.

*P < 0.05; **P < 0.01.

CDSS = Calgary Depression Scale for Schizophrenics; SANS = Scale for the Assessment of Negative Symptoms.

For the total schizophrenic sample, PAS and SAS total scores were significantly and positively correlated with negative symptoms (SANS total score (*P *< 0.01), SANS 'affective flattening' subscale score (*P *< 0.05), and SANS 'anhedonia/asociality' subscale score (*P *< 0.01)) and disorganisation (*P *< 0.01) (particularly with the SAPS 'bizarre behaviour' subscale score (*P *< 0.05) and the SAPS 'formal thought disorders' subscale score (*P *< 0.05)). No correlations with positive dimension and depressive symptoms were detected (Table 3).

**Table 3:** Spearman correlation coefficients between anhedonia scores and general psychopathological variables in the total schizophrenic sample (n = 80)

**Psychopathological variables**	**PAS total score**	**SAS total score**
Negative dimension (SANS total score)	0.37**	0.34**
Affective flattening	0.13	0.29*
Alogia	0.12	0.04
Avolition/apathy	0.14	0.1
Anhedonia/asociality	0.38**	0.37**
Positive dimension	-0.01	0.14
Hallucinations	-0.09	0.13
Delusions	0.06	0.15
Disorganised dimension	0.36**	0.35**
Bizarre behaviour	0.16	0.26*
Formal thought disorders	0.27*	0.28*
Attentional impairment	0.14	0.13
Depression (CDSS total score)	-0.05	0.08

Spearman correlation coefficient (R) values are reported.

*P < 0.05; **P < 0.01.

CDSS = Calgary Depression Scale for Schizophrenics; PAS = Physical Anhedonia Scale; SANS = Scale for the Assessment of Negative Symptoms; SAS = Social Anhedonia Scale.

### Depressed patients

The mean (SD) anhedonia scores for the depressed sample were 15.32 ± 6.72 for physical anhedonia (PAS total score) and 13.07 ± 5.66 for social anhedonia (SAS total score) (Table 1). Of the analysed depressed subjects, 27 (41.5%) reached or passed the PAS cut-off, 32 (49.2%) the SAS cut-off and 24 (36.9%) the 'double cut-off' (Table 1).

The comparison for general psychopathological parameters between 'anhedonic' and 'normal hedonic' depressed subgroups revealed that the former displayed higher levels of clinical depression (HDRS total score (*P *< 0.05)) and negative symptoms (SANS total score (*P *< 0.05) and SANS 'alogia' subscale score (*P *< 0.01)) (Table 4). No differences were detected between depressed subgroups in terms of gender, civil state, occupation, age, years of education, duration of illness, type and dosage of medication (selective serotonin reuptake inhibitors (SSRIs)/non-selective serotonin reuptake inhibitors (NSRIs) vs tricyclic antidepressant drugs).

**Table 4:** Comparison of general psychopathological parameters between 'anhedonic' and 'normal hedonic' depressed patients

**Psychopathological variables**	**'Normal hedonic' depressed patients (n = 41)**	**'Anhedonic' depressed patients (n = 24)**	**Z value**
Depression (HDRS total score)	14.93 ± 4.84	19.50 ± 7.48	-2.63*
Negative dimension (SANS total score)	19.37 ± 15.07	31.67 ± 17.17	-2.76*
Affective flattening	6.00 ± 5.67	9.83 ± 8.01	-1.69
Alogia	1.83 ± 3.25	5.17 ± 5.02	-3.44**
Avolition/apathy	3.97 ± 3.28	5.92 ± 2.78	-1.41
Anhedonia/asociality	7.57 ± 5.15	9.75 ± 4.94	-1.39

Mean ± standard deviation (SD) and Mann-Whitney Z test values are reported.

*P < 0.05; **P < 0.01.

HDRS = Hamilton Depression Rating Scale; SANS = Scale for the Assessment of Negative Symptoms.

In the total depressed sample, PAS and SAS total scores were significantly and positively correlated with clinical depression severity (HDRS total score (*P *< 0.01)) and negative symptoms (SANS total score (*P *< 0.01), SANS 'alogia' subscale score (*P *< 0.05), SANS 'avolition/apathy' subscale score (*P *< 0.01), and SANS 'anhedonia/asociality' subscale score (*P *< 0.01)) (Table 5).

**Table 5:** Spearman correlation coefficients between anhedonia scores and general psychopathological variables in the total depressed sample (n = 65)

**Psychopathological variables**	**PAS total score**	**SAS total score**
Depression (HDRS total score)	0.42**	0.39**
Negative dimension (SANS total score)	0.40**	0.37**
Affective flattening	0.18	0.17
Alogia	0.27*	0.18
Avolition/apathy	0.44**	0.41**
Anhedonia/asociality	0.38**	0.43**

Spearman correlation coefficient (R) values are reported.

*P < 0.05; **P < 0.01.

HDRS = Hamilton Depression Rating Scale; PAS = Physical Anhedonia Scale; SANS = Scale for the Assessment of Negative Symptoms; SAS = Social Anhedonia Scale.

## Discussion

### Schizophrenic patients

In accord with several authors [5,8,14,52-54], our results reveal that anhedonia reaches clinically significant levels only in a subgroup of schizophrenic patients (45% of the total psychotic sample) (Table 1). These findings suggest that the Meehl's hypothesis of anhedonia linked to schizophrenia by an etiopathogenetical tie of necessity [18,55] does not seem to be legitimated in all schizophrenic subjects, but at most it seems to concern exclusively the schizophrenic group characterised by high levels of negative symptoms and disorganisation (that is, negative, deficit, and hebephrenic subtypes) (Table 2).

The higher levels of negative symptoms in the 'anhedonic' schizophrenic subgroup do not seem to be traced back to the SANS 'anhedonia/asociality' subscale load, because its scores show no statistically significant differences between 'anhedonic' and 'normal hedonic' schizophrenics (Table 2). According to many authors [51,56,57], these data reveal the psychometric discrepancy between anhedonia self-report questionnaires (that is, PAS and SAS scales) and anhedonia interview-based inventories (that is, SANS), as well as the unreliability of the latter instruments in measuring the real hedonic ability in schizophrenic subjects. Thus, even if the 'anhedonia' psychopathological construct can be confused (because of its 'minus' clinical features) with a SANS negative symptom, it does not really seem to identify with the SANS 'anhedonia/asociality' subscale.

**Figure 1:**
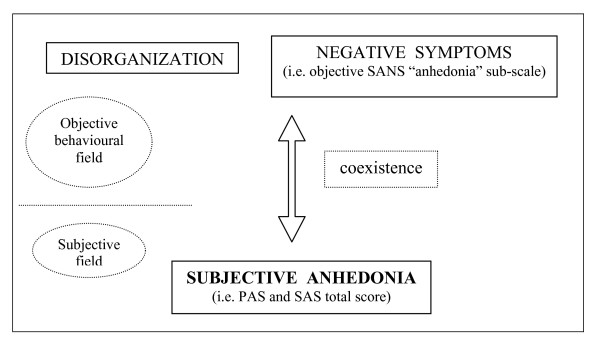
**Psychopathological relations among anhedonia, disorganisation, and negative symptoms in schizophrenia**.

The positive correlation of subjective anhedonia (PAS and SAS total scores) with negative symptoms (Table 3) suggests a clinical coexistence of hedonic deficit and negative symptoms of schizophrenia. Anhedonia measured by the Chapman self-report scales (subjective anhedonia) could represent a subjective psychopathological experience which coexists and comes together with some of the negative behavioural components obtained by the SANS (that is, objective 'affective flattening' and 'anhedonia/asociality' subscales) (Figure 1).

In accord with Loas *et al*. [58], our results reveal that 'anhedonic' schizophrenics also show higher levels of disorganisation than 'normal hedonic' schizophrenics (Table 2). The positive correlation of subjective anhedonia (PAS and SAS total scores) with disorganised symptoms (Table 3) reveals a clinical coexistence of hedonic deficit and schizophrenic disorganisation. Anhedonia estimated by the Chapman self-report scales (subjective anhedonia) could also represent a subjective psychopathological experience which coexists and accompanies the schizophrenic behavioural disorganisation measured by the SANS and the SAPS (Figure 1). Those findings appear partially to agree with the conclusions suggested by Loas *et al*. [58], who have considered the anhedonic symptomatology of disorganised chronic schizophrenics as a specific symptom of their psychotic chronicity.

The lack of different levels of depression and positive symptoms in 'anhedonic' and 'normal hedonic' schizophrenics (Table 2) and the absence of significant correlations between anhedonia (PAS and SAS total scores) and depressive or positive dimensions (Table 3) suggest the psychopathological independence of hedonic deficit from depression and 'psychoticism' (hallucinations and delusions).

### Depressed patients

In accord with several authors [27-29], our results reveal that anhedonia reaches clinically significant levels only in a subgroup of depressed patients (36.9% of the total depressed sample) (Table 1). These findings suggest that the Loas's hypothesis of anhedonia linked to major depression by an etiopathogenetical tie of necessity [39] does not seem to be legitimate in all depressed subjects, but at most it seems exclusively to be a specific psychopathological marker of those major depressive forms (subtypes) which present a marked clinical depression severity (that is, 'endogenomorphic', 'melancholic' or 'vital' syndromes) and higher HDRS total scores (Table 4). Furthermore, the high levels of anhedonia found in most of our schizophrenic patients seem to suggest that the DSM-IV-TR criteria to consider the hedonic deficit as a distinctive state-like symptom of major depression [30] does not match the clinical reality.

**Figure 2:**
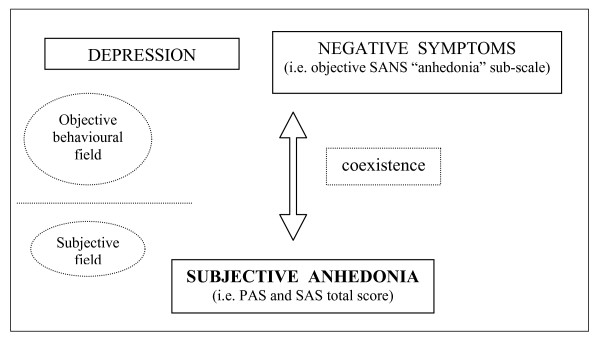
**Psychopathological relations among anhedonia, clinical depression, and negative symptoms in major depressive disorder**.

The positive correlation of subjective anhedonia (PAS and SAS total scores) with depressive symptoms (HDRS total score) (Table 5) reveals a clinical coexistence of hedonic deficit and the severity of major depression psychopathology. Anhedonia estimated by the Chapman self-report scales (subjective anhedonia) could represent a subjective psychopathological experience that coexists and comes together with the objective behavioural depressive symptoms measured by the HDRS (Figure 2).

In accord with Joiner *et al*. [32], our results also reveal that 'anhedonic' depressed patients show higher levels of negative symptoms than 'normal hedonic' subjects (Table 4). This finding does not seem to be traced back to the SANS 'anhedonia/asociality' subscale load, because its scores show no statistically significant differences between 'anhedonic' and 'normal hedonic' depressed subgroups. Otherwise, these data reveal the psychometric discrepancy between anhedonia self-report questionnaires (that is, PAS and SAS scales) and anhedonia interview-based rating scales (that is, SANS) [51], as well as the unreliability of the latter instruments in measuring the real hedonic deficit in depressed patients [44,52].

The positive correlation of subjective anhedonia (PAS and SAS total scores) with negative symptoms (Table 5) also suggests a clinical coexistence of hedonic deficit and negative symptoms of major depressive disorder. Anhedonia measured by the Chapman self-report scales (subjective anhedonia) could also represent (as well as for the depressive symptoms) a subjective psychopathological experience that coexists and accompanies the negative behavioural components obtained by the SANS (that is, objective 'alogia', 'avolition/apathy', and 'anhedonia/asociality' subscales) (Figure 2).

## Conclusion

### Schizophrenic patients

The results of this study reveal that anhedonia reaches clinically significant levels only in a subgroup of schizophrenic patients (45%), in which it entertains strong psychopathological relations with negative and disorganised dimensions. In other words, hedonic inability seems to be a specific subjective psychopathological experience of those schizophrenic forms characterised by a marked severity of negative symptoms (that is, 'negative' or 'deficit' syndromes) and cognitive/behavioural disorganisation (that is, 'hebephrenic' type).

**Figure 3:**
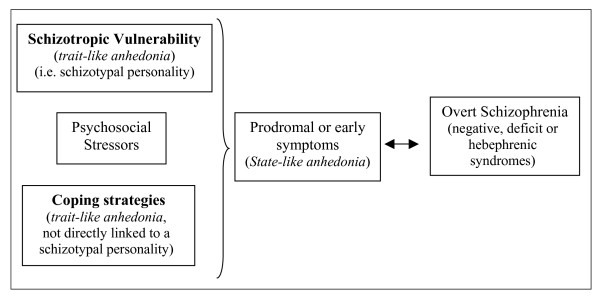
**Possible positions of anhedonia in the 'vulnerability/stress/coping model' of schizophrenia (Zubin *et al*.) **[59].

According to the 'vulnerability/stress/coping model' of schizophrenia proposed by Zubin *et al*. [59], it can be hypothesised that the subjective 'enduring' features of anhedonia estimated by the Chapman self-report scales could play the role assigned to prodromal or early symptoms of a schizophrenic psychosis (subjective state-like anhedonia) particularly for the negative, deficit or disorganised subtypes, or that they could be one of the schizotropic vulnerability factors of a prepsychotic personality (subjective trait-like anhedonia). As an alternative, the subjective hedonic deficit could be considered as a negative personological trait that increases the probability of psychotic decompensation of a prepsychotic temperament (using disadaptative coping strategies), without being a direct characterial index of a schizophrenic vulnerability (Figure 3).

### Depressed patients

The results of this study reveal that anhedonia reaches clinically significant levels only in a subgroup of depressed patients (36.9%), where it entertains strong psychopathological relations with negative and depressive symptoms. In other words, hedonic inability seems to be a specific subjective psychopathological experience of those major depressive forms characterised by a marked clinical depression severity and higher HDRS and SANS total scores (that is, 'melancholic', 'endogenomorphic' or 'vital' depressive subtypes).

**Figure 4:**
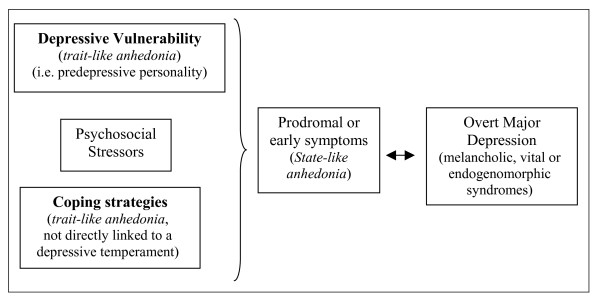
**Possible positions of anhedonia in Loas's 'vulnerability to major depression model' (Loas) **[39].

According to the 'vulnerability to depression model' proposed by Loas [39], it can be hypothesised that the subjective enduring features of anhedonia evaluated by the Chapman self-report scales could play the role assigned to prodromal or early symptoms of depressive disorder (subjective state-like anhedonia) (particularly for the melancholic, vital or endogenomorphic syndromes) or that they could be one of the vulnerability factors of a predepressive personality (subjective trait-like anhedonia). As an alternative, the subjective hedonic deficit could be considered as a negative personological trait that increases the probability of clinical decompensation of a depressive temperament (using disadaptative coping strategies), without being a direct characterial index of a depressive vulnerability (Figure 4).

At the very least, we should mention some limitations of this study. First, our schizophrenic and major depressed samples were composed only of outpatients in maintenance treatment and by a mixed population of subjects regarding their pharmacological status and longitudinal course (that is, 'single' vs 'recurrent' depressive episodes). Thus, further studies (including inpatient samples and a more selective population in terms of medication and duration of illness) to elucidate the real nature of anhedonia in schizophrenia and major depression are needed. Moreover, our depressed sample was numerically quite small (n = 65). Thus, further studies in a larger depressed population are needed.

Furthermore, in this study, to rate hedonic capacity we used the Chapman scales for physical and social anhedonia (PAS and SAS), two validated self-report instruments measuring the subjective enduring features of hedonic inability to experience a wide range of sensory and interpersonal pleasures (such as eating, touching, sex, smell, and sound) [5]. However, recently, some authors [42,43] have suggested that the Snaith Hamilton Pleasure Scale (SHAPS) [60] is a more appropriate instrument to evaluate hedonic ability, considering it the golden standard to rate anhedonia. Thus, further studies using SHAPS to confirm and replicate our results are needed.

Finally, we want to underline a limitation regarding the application of Spearman correlations in a cross-sectional study. This statistical method reflects exclusively a coexistence of anhedonia and negative symptoms or disorganisation in schizophrenics, and a coexistence of hedonic deficit and depression in major depressive disorder. Thus, to confirm and demonstrate the possible positions of anhedonia proposed in Figures 3 and 4, further prospective and longitudinal studies are needed.

## Competing interests

The authors declare that they have no competing interests.

## Authors' contributions

Both LP and AF participated in the design of the study and in the acquisition of data, performed the statistical analysis and helped to draft the manuscript.
